# Effects of Probiotics, Prebiotics, and Synbiotics on Uremic Toxins, Inflammation, and Oxidative Stress in Hemodialysis Patients: A Systematic Review and Meta-Analysis of Randomized Controlled Trials

**DOI:** 10.3390/jcm10194456

**Published:** 2021-09-28

**Authors:** Thi Thuy Uyen Nguyen, Hyeong Wan Kim, Won Kim

**Affiliations:** 1 Department of Histology, Embryology, Pathology and Forensic Medicine, Hue University of Medicine and Pharmacy, Hue University, Hue 52000, Vietnam; nttuyen@hueuni.edu.vn; 2 Department of Internal Medicine, Jeonbuk National University Medical School, Jeonju 54896, Korea; ocaju78@hanmail.net; 3 Research Institute of Clinical Medicine of Jeonbuk National University-Biomedical Research Institute of Jeonbuk National University Hospital, Jeonju 54907, Korea

**Keywords:** probiotic, prebiotic, synbiotic, hemodialysis, chronic kidney disease

## Abstract

The dysbiosis of gut microbiota may cause many complications in patients with end-stage renal disease, which may be alleviated by probiotic, prebiotic, and synbiotic supplementation. The aim of this systematic review and meta-analysis was to assess the effects of these supplementations on circulatory uremic toxins, biomarkers of inflammation, and oxidative stress in hemodialysis patients. We searched the EMBASE, MEDLINE, Web of Science, and Cochrane Library databases until 8 August 2021. Randomized controlled trials evaluating adult patients receiving hemodialysis were included. The pooled results from 23 studies with 931 hemodialysis patients indicated that interventions significantly decreased the circulating levels of p-cresyl sulfate (standardized mean difference (SMD): 0.38; 95% CI: −0.61, −0.15; *p* = 0.001), endotoxins (SMD: −0.58; 95% CI: −0.99, −0.18; *p* = 0.005), malondialdehyde (SMD: −1.16; 95% CI: −1.81, −0.52; *p* = 0.0004), C-reactive proteins (CRP) (SMD: −0.61; 95% CI: −0.99, −0.23; *p* = 0.002), and interleukin 6 (SMD: −0.92; 95% CI: −1.51, −0.33; *p* = 0.002), and improved the total antioxidant capacity (SMD: 0.89; 95% CI: 0.49, 1.30; *p* < 0.0001) and glutathione (SMD: 0.40; 95% CI: 0.14, 0.66; *p* = 0.003) when compared to the placebo group. Our results suggest that treatment with probiotics, prebiotics, and synbiotics may help alleviate uremic toxin levels, oxidative stress, and the inflammatory status in hemodialysis patients.

## 1. Introduction

The dysbiosis of gut microbiota due to an increased urea secretion into the digestive system contributes to circulating uremic toxins, systemic inflammation, oxidative stress, cardiovascular events, and other complications in patients with end-stage renal disease (ESRD) [1,2]. Although hemodialysis is an advanced kidney replacement therapy, the morbidity and mortality remain unacceptable [3]. Protein-binding toxins, such as p-cresyl sulfate (p-CS) and indoxyl sulfate (IS), cannot be completely eliminated by hemodialysis [4]. Protecting the imbalance of the intestinal microbiome may be a promising way to improve outcomes in hemodialysis patients [5].

Targeted therapies to restore symbiosis have been suggested to alleviate systemic symptoms and improve renal outcomes. Among them, probiotics are beneficial living microorganisms that help to balance the intestinal microbiota profile [6,7]. Prebiotics are non-digestible substrates that play an essential role in enhancing the development of beneficial gut microorganisms, including resident microorganisms and probiotic strains [8]. Synbiotics are combinations of probiotics and prebiotics that can synergistically affect the gastrointestinal tract [9,10].

Several meta-analyses have evaluated the effects of probiotics, prebiotics, and synbiotics in patients with decreased renal function. A systematic review and meta-analysis demonstrated that probiotic supplementation reduced inflammation and uremic toxin levels and improved gastrointestinal symptoms in patients undergoing hemodialysis or peritoneal dialysis. However, this review pooled the data after the administration of probiotics from both non-randomized control trials and randomized control trials [11]. A meta-analysis that focused on their effects in chronic kidney disease (CKD) patients with or without dialysis has been reported [12]. Recently, March et al. demonstrated their efficacy on gut-derived toxic metabolites, lipid profiles, and clinical outcomes in patients receiving hemodialysis or peritoneal dialysis [13].

Dialysis modalities, such as hemodialysis and peritoneal dialysis, may affect uremic toxin levels and oxidative stress. Therefore, the effects of probiotic, prebiotic, and synbiotic supplementation on uremic toxin levels and oxidative stress may be different when combined with different dialysis modalities. To our knowledge, no meta-analysis has intensively assessed their effects on uremic toxins, inflammation, and oxidative stress in patients with hemodialysis alone. In particular, their effects on the oxidative stress status have not been reviewed.

Therefore, our systematic review and meta-analysis was performed to evaluate results from the available randomized controlled trials to assess the benefits of probiotics, prebiotics, and synbiotics on the oxidative stress status and confirm their overall effects on circulating uremic toxins, endotoxins, and inflammation among hemodialysis patients.

## 2. Materials and Methods

### 2.1. Registration and Protocol

This systematic review and meta-analysis was registered in the International Prospective Register of Systematic Review (PROSPERO) database (Registration No. CRD42021246823) and conducted according to the Preferred Reporting Items for Systematic Reviews and Meta-Analysis Statement (PRISMA) guidelines [14].

### 2.2. Eligibility Criteria

The eligible studies for inclusion in this review were chosen according to the PICOS framework. (1) Participants: adult ESRD patients with regular hemodialysis were enrolled; (2) Intervention: probiotic, prebiotic, or synbiotic supplementation; (3) Comparison: placebo or any vehicle containing the same format as the intervention, but without experimental active components; (4) Outcome: the primary outcomes were the levels of uremic toxins (p-CS, IS) and endotoxins, whereas the secondary outcomes were biomarkers of inflammation (C-reactive protein [CRP], inteulerkin 6 [IL-6]), and oxidative stress status (malondialdehyde [MDA], total antioxidant capacity [TAC], glutathione [GSH]); and (5) Study design: randomized controlled trials. All studies were published in English, without any restrictions on the year of dissemination. Studies were excluded if the outcome-of-interest was not evaluated, and studies that did not satisfy the inclusion criteria were excluded.

### 2.3. Information Sources and Search Strategy

A comprehensive literature search for this review was conducted on four electronic databases: EMBASE, MEDLINE, Web of Science, and Cochrane Library, until August 8, 2021. The electronic search used the text and MeSH terms: “probiotics”, “prebiotics”, “dietary fiber”, “resistant starch”, “synbiotics”, and “hemodialysis”. For better readability, the full search strategy is outlined in the Table A1. The duplicate results of the four databases were cross-checked and eliminated using Endnote X9 (Clarivate Analytics, Philadelphia, PA). A secondary search for relevant studies was performed using references from the included studies. Two investigators (T.T.U.N. and H.W.K.) conducted and assessed this search strategy.

### 2.4. Study Selection and Data Collection

We assessed the publications based on predetermined eligibility criteria and summarized the study collection processes using the PRISMA flow diagram. The full-text articles were examined after excluding irrelevant titles and abstracts by two independent investigators (T.T.U.N. and H.W.K.), and any disagreements were resolved by discussion and consensus with the third author (W.K.). We created a data extraction template using Microsoft Office Excel 2010 to collect numerical data from the included studies. One author extracted the data, and the other author checked. In the case of divergent decisions, we resolved by consensus of two authors or by discussion with the third author. In several trials, the numerical data for meta-analysis were only provided by the figure. In that case, we requested the data from the corresponding author or used WebPlotDigitizer version 4.4 (https://automeris.io/WebPlotDigitizer/ accessed on 18 June 2021) to extract the numerical data from figures.

### 2.5. Data Items

From the included studies, the data were independently collected by two authors (T.T.U.N and H.W.K.) according to study source (study name, authors, year of publication, and country), characteristics of study and population (study design, sample size, proportion of men/women, mean age, mean body mass index (BMI), and hemodialysis duration), groups of trials (number of patients in each group, type, species, dosage, and duration of intervention), and the data of outcomes. In a randomized controlled crossover study, we extracted the final evaluation data.

### 2.6. Risk of Bias Assessment

We used the Cochrane risk-of-bias tool for randomized trials version 2 (RoB2) structured into five domains to assess the quality of the included RCTs [15]. “Low risk of bias” or “some concerns” or “high risk of bias” were the risk-of-bias decisions for each domain. Quality assessments were independently conducted by two authors (T.T.U.N and H.W.K.), and any disagreements were resolved by discussion and consensus with the third author (W.K.).

### 2.7. Data Analysis

All extracted data were continuous data and are presented as mean and standard deviation (SD). If the trial authors only provided data in median and interquartile ranges or mean with 95% confidence interval, we estimated means and SDs using the formula of Wan et al. or Cochrane’s recommendation, chapter 6.5.2, respectively [16,17]. In this meta-analysis, we used Review Manager (RevMan) (Computer program) (Version 5.4, The Cochrane Collaboration (https://training.cochrane.org/online-learning/core-software-cochrane-reviews/revman/revman-non-cochrane-reviews (accessed on 15 August 2021)), 2020) based on standardized mean difference (SMD) for summarizing statistics of continuous data with different measurements or units. Our analysis was based on the means and SDs of the changes from baseline score. When the SDs of mean differences were not provided in the trial, we estimated SDs based on the recommended formula from chapter 6.5.2.8 of the *Cochrane Handbook* [17]. If there were two interventions compared with one control group in one trial, we split the control group in half to avoid counting twice. The I-squared statistic (I^2^) was used to assess heterogeneity. I^2^ ≤ 40% and *p*-value ≥ 0.1 among studies were considered to have low heterogeneity, and a fixed-effects model was used for estimating. In any case, a random-effects model was used. Heterogeneity was defined as moderate (40% < I^2^ ≤ 70%) or high (I^2^ > 70%) [18]. If the meta-analysis included 10 or more studies, we assessed publication bias via funnel plot asymmetry using RevMan 5.4 software [19]. Egger’s regression test in Comprehensive Meta-Analysis (CMA) version 2.0 software was the statistical test for funnel plot asymmetry. All tests, except for the heterogeneity test, received a statistical significance of *p* < 0.05.

## 3. Results

### 3.1. Search and Selection of Studies

**Figure 1:**
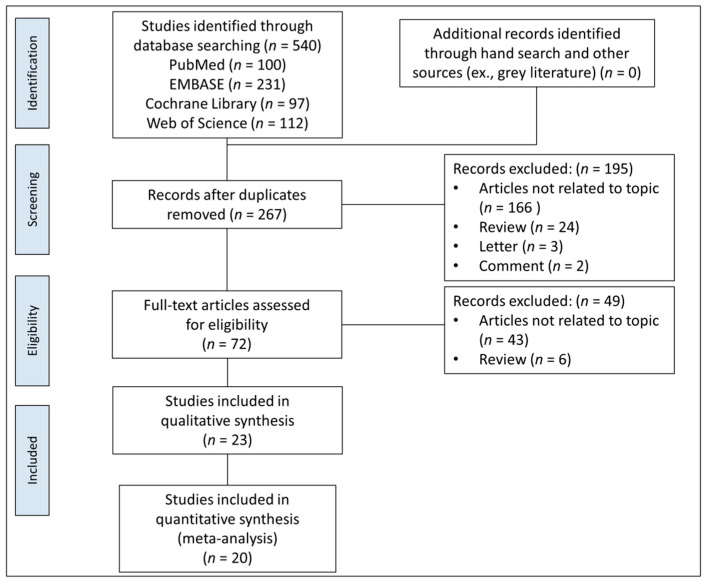
PRISMA flow diagram for identification of relevant studies.

After searching four electronic databases and relevant bibliographies, 540 articles were obtained, after which, 267 unduplicated studies were screened for titles and abstracts, and 195 articles were discarded. We reviewed the full texts of the remaining 72 articles, and 49 were excluded according to the predetermined criteria (Figure 1). Finally, 23 studies were eligible for qualitative synthesis, and 20 were selected for quantitative analysis.

### 3.2. Characteristics of Included Studies

As presented in Table 1, all 23 articles from 20 RCTs were published in English between 2014 and 2021. Eleven of them were conducted in Iran (48%), six studies in Brazil (26%), two each in the USA (9%) and China (9%), and only one in Mexico and Taiwan (4%).

All RCTs investigated the efficacy of probiotics, prebiotics, and synbiotics in 931 adult hemodialysis patients. Seven studies assessed probiotics, eight studies assessed prebiotics, six studies assessed synbiotics, and two studies used both probiotics and synbiotics for interventions. The hemodialysis duration of all participants with mean age ranging from 32 to 63 years is presented in Table 1. The shortest duration of intervention was 4 weeks [20,21,22,23], and the longest was 24 weeks [24,25].

### 3.3. Quality Assessment

**Figure 2:**
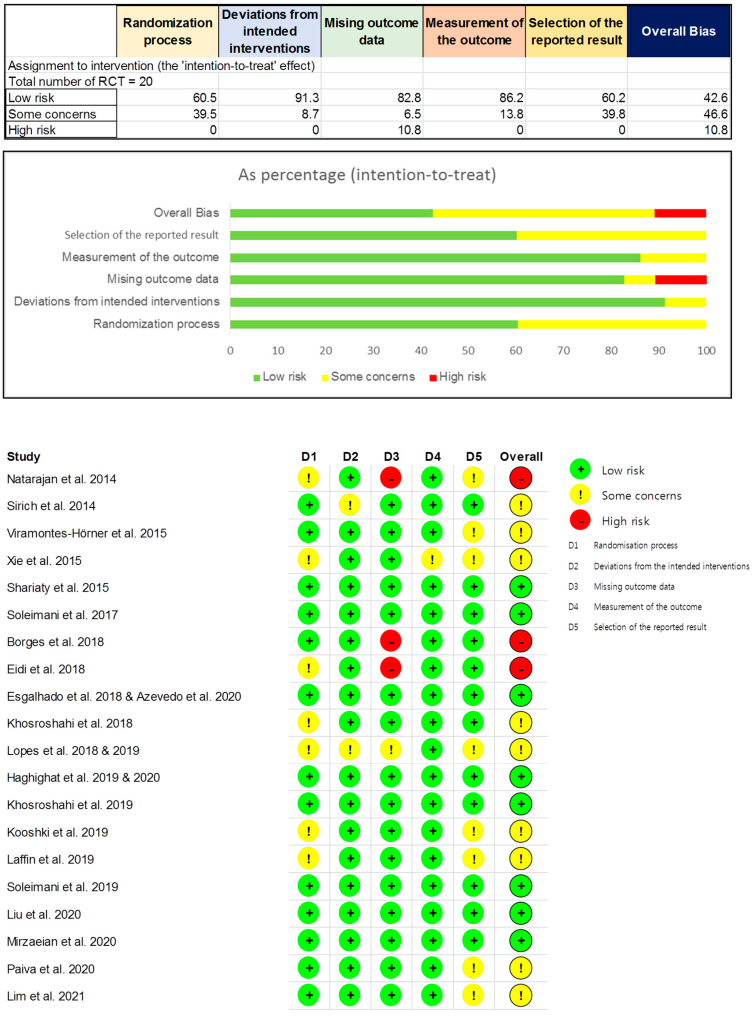
Risk of bias assessment for included RCTs using version 2 of Cochrane risk-of-bias tool for randomized trials.

According to the Cochrane risk-of-bias tool for randomized trials version 2, eight RCTs were assessed as “low risk of bias” (42.6%), nine RCTs as “some concerns” (46.6%), and three RCTs as “high risk of bias” (10.8%) (Figure 2).

### 3.4. Meta-Analysis

#### 3.4.1. Effects on Circulating Uremic Toxins

**Figure 3:**
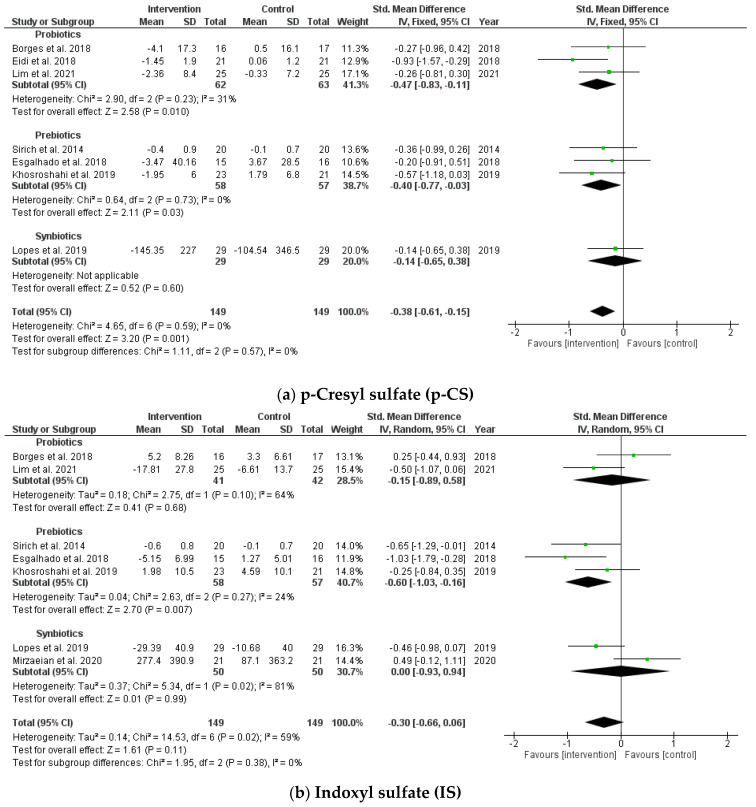
Forest plot for probiotic, prebiotic, and synbiotic effects on (**a**) p-cresyl sulfate (p-CS) and (**b**) indoxyl sulfate (IS) in hemodialysis patients.

In this study, the pooled analysis showed a significant decrease in circulating uremic toxin, p-CS, after taking probiotics, prebiotics, and synbiotics (SMD = −0.38; 95% CI: −0.61, −0.15; *p* = 0.001, I^2^ = 0%). However, no significant differences were found after supplementation between the intervention and placebo groups on IS (SMD = −0.30; 95% CI: −0.66, 0.06; *p* = 0.11, I^2^ = 59%) (Figure 3). No publication bias was observed in the funnel plot and Egger’s test for p-CS and IS (*p* = 0.643 and *p* = 0.884, respectively).

#### 3.4.2. Effects on Endotoxins

**Figure 4:**
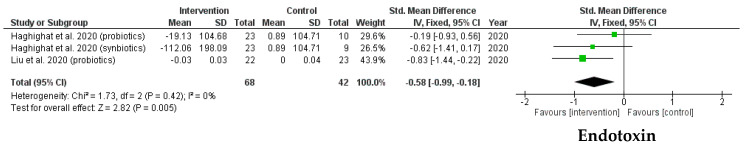
Forest plot for probiotic, prebiotic, and synbiotic effects on endotoxins in hemodialysis patients.

A forest plot comparing the SMD of endotoxins is shown in Figure 4. The results indicated a significantly positive effect in the probiotic, prebiotic, and synbiotic groups (SMD = −0.58; 95% CI: −0.99, −0.18; *p* = 0.005, I^2^ = 0%). In addition, the heterogeneity of the SMDs was considered low.

#### 3.4.3. Effects on Biomarkers of Oxidative Stress

**Figure 5:**
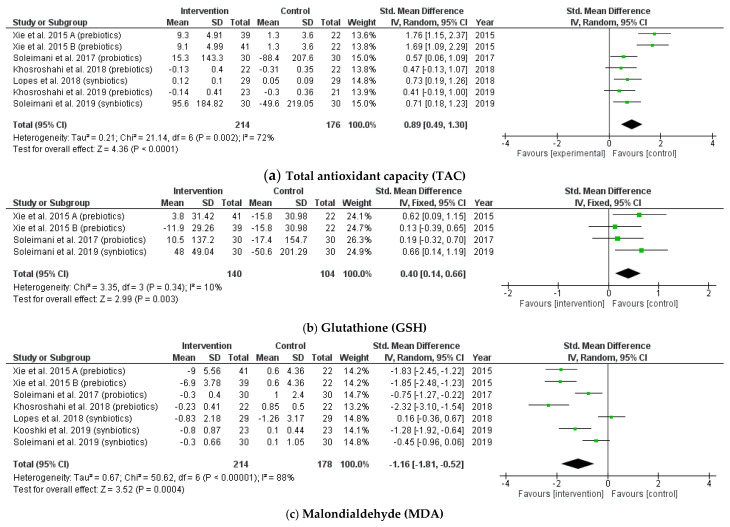
Forest plot for probiotic, prebiotic, and synbiotic effects on oxidative stress status: (**a**) total antioxidant capacity (TAC); (**b**) glutathion (GSH); (**c**) malondialdehyde (MDA) in hemodialysis patients.

Regarding biomarkers of the oxidative stress status, the pooled analysis indicated significant changes in TAC levels following probiotic, prebiotic, and synbiotic supplementation (SMD = 0.89; 95% CI: 0.49, 1.30; *p* < 0.0001, I^2^ = 72%). A remarkable increase in GSH was found in the intervention group with low heterogeneity among trials (SMD = 0.40; 95% CI: 0.14, 0.66; *p* = 0.003, I^2^ = 10%). In addition, there was a reduction in MDA levels in the intervention group when compared with the placebo group, as shown in Figure 5. (SMD = −1.16; 95% CI: −1.81, −0.52; *p* = 0.0004, I^2^ = 88%).

#### 3.4.4. Effects on Biomarkers of Inflammation

**Figure 6:**
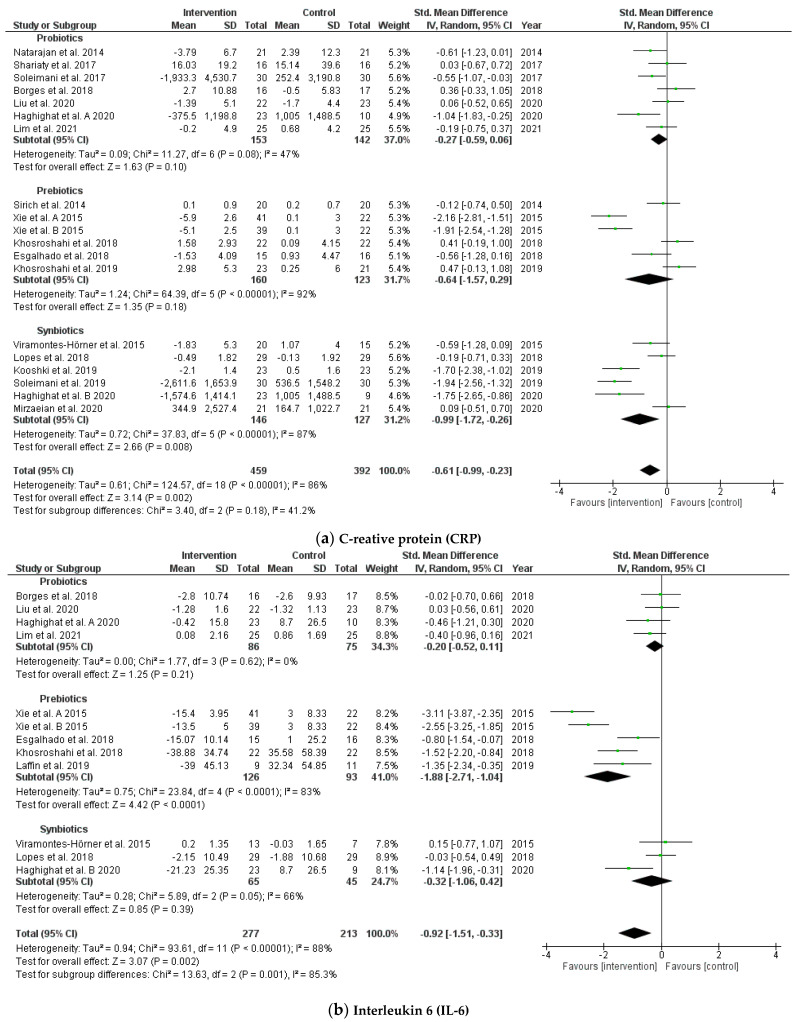
Forest plot for probiotic, prebiotic, and synbiotic effects on biomarkers of inflammation: (**a**) C-reative protein (CRP); (**b**) interleukin 6 (IL-6) in hemodialysis patients.

The pooled results of 19 RCTs indicated that probiotic, prebiotic, and synbiotic supplementation contributed to reduced CRP levels (SMD = −0.61; 95% CI: −0.99, −0.23; *p* = 0.002, I^2^ = 86%) and IL-6 (SMD = −0.92; 95% CI: −1.51, −0.33; *p* = 0.002, I^2^ = 88%) among patients receiving hemodialysis (Figure 6). The heterogeneity of the SMDs was considered to be high in inflammation biomarkers. The funnel plot and Egger’s test showed no publication bias for CRP and IL-6 (*p* = 0.178 and *p* = 0.233, respectively).

## 4. Discussion

In this review, we summarized the results from randomized controlled trials and assessed the efficacy of probiotics, prebiotics, and synbiotics on uremic toxins, endotoxins, inflammation, and the oxidative stress status in hemodialysis patients. Our main findings indicated that these could significantly reduce the levels of circulating toxins (p-CS, endotoxins) and biomarkers of inflammation (CRP, IL-6), and ameliorate the balance between antioxidant and pro-oxidant markers (TAC, GSH, and MDA) when compared with the intervention and placebo groups.

This meta-analysis found reductions in p-CS and endotoxin levels after supplementation. This is consistent with the conclusion of a previous review by March et al., which included trials with patients receiving hemodialysis or peritoneal dialysis [13]. However, in contrast to our findings, they reported that probiotic, prebiotic, and synbiotic supplementation had a remarkable effect on decreasing IS levels. The difference can be explained by the method of data collection and the number of included studies. A previous review conducted an analysis based on the post-intervention data, whereas we collected the means and SDs of the changes between the baseline and final values, which yielded a significant difference in the pooled IS data of Borges et al.’s trial [32]. Notably, three recent trials have been added to our assessment [21,25,42]. It is worth noting that, in a subgroup review of studies that focused on intervention therapy, we discovered that prebiotics have a significant effect on reducing IS. The number of trials that focus on the effects of probiotics or synbiotics on the level of IS in hemodialysis patients is still limited (two and two trials, respectively). Thus, further studies are necessitated.

In line with a previous study by Zheng et al., we found that serum hs-CRP levels were significantly reduced in the intervention group [43]. However, the results were pooled from CKD patients receiving and not receiving dialysis. Our results were obtained from patients undergoing hemodialysis. Therefore, there was a difference in the target patients. Although our pooled results indicated that heterogeneities were considered as high in the assessment of inflammation biomarkers between studies, we report this result and recommend further high-quality studies with a consensus on the methodology in order to solve these inconsistent results.

Oxidative stress can be found even in the early stages, progresses with renal failure, and is exacerbated by the hemodialysis process. In addition, excessive oxidative stress and inflammation contribute to increased cardiovascular disease and an accelerated mortality in ESRD [44,45]. The pooled analyses of 13 clinical trials in CKD patients showed that prebiotics, probiotics, and synbiotics can reduce the oxidative stress status by ameliorating the oxidative activity (MDA) and improving the antioxidant capacity and enzymes (TAC and GSH) [43]. Nevertheless, there has not been an intensive report assessing the effectiveness of these supplements in patients receiving maintenance hemodialysis alone. This is the first meta-analysis to show their benefits on the oxidative stress status in hemodialysis patients. Although the heterogeneity was considerable among trials reporting the assessment of MDA, six out of seven studies indicated a change in MDA after intervention.

In patients with CKD, uremic toxins increase the affinity for binding proteins and cannot be excreted through renal proximal tubules. This may be related to the imbalance of gut microbiota, leading to increased uremic toxins, the activation of pro-inflammation, metabolic disorders, oxidative stress, and aggravating the progression of kidney failure [10,46,47,48]. For decades, probiotics have been determined to create a healthy gut microbiome community, which is also the first and foremost factor in the complex mechanism of probiotics in kidney failure [10]. To date, we have confirmed that probiotics, prebiotics, and synbiotics may be effective in alleviating disorders associated with circulating toxins, chronic inflammation, and oxidative stress in hemodialysis patients.

There were some limitations to the present meta-analysis. Although we showed that these have significant effects when compared with the intervention and control groups, the heterogeneity in the pooled results was statistically significant among the included studies that assessed the circulating levels of CRP, IL-6, TAC, and MDA. We conducted the subgroup analyses according to the duration of intervention. However, due to the limited number of studies that used probiotics, prebiotics, and synbiotics in a longer intervention, we have not been able to identify the solid results of the subgroup analysis. Additionally, we have not been able to compare the effectiveness of various probiotic and prebiotic strains used in clinical trials. Hence, it was difficult to provide therapeutic recommendations for clinicians because of the diversity of probiotics and prebiotics, as well as the difference in dosage and duration of consumption. Due to the limited number of trials in each type of supplementation, we did not specifically evaluate their effects on all biomarkers in hemodialysis patients. The number of studies with a high risk of bias is not large; however, there are still many studies that receive “some concerns” reviews. Therefore, larger trials with higher quality are needed.

Upon assessing the included RCTs, we identified some deficiencies in the methodology and reporting. Hence, these recommendations on the conduct and reporting may enhance the quality of RCTs: using random components for the sequence generation process, concealing the allocation sequence to participants and assessors, avoiding deviations from intended interventions, correcting bias due to missing outcome data, and avoiding bias in the selection of reported results (completing analyses in accordance with pre-specified trial protocol and analysis plan before unblinded outcome data are available).

## 5. Conclusions

Probiotics, prebiotics, and synbiotics not only have significant effects on the reduction in p-CS and endotoxins, but also have remarkable benefits in rebalancing the oxidative status in patients receiving maintenance hemodialysis.

## Figures and Tables

**Table 1:** Characteristics of included reports in systematic review.

Author, Year	Country	Type of Study	Population (M/F)	Mean Age (Years)	Mean BMI (kg/m^2^)	Hemodialysis	Intervention Group (*n*)	Control Group (*n*)	Outcome	Duration
Natarajan et al., 2014 [26]	USA	Randomized, double-blind, placebo-controlled crossover trial	22 (16/8)	54	NA	NA	Probiotics: L. acidophilus KB 27, S. thermophilus KB 19, and B. longum KB 31 (*n* = 22)	Placebo (*n* = 22)	Hs-CRP, TIG	2 months
Sirich et al., 2014 [27]	USA	Randomized, single-blind, placebo-controlled clinical trial	40 (24/16)	56	29	HD, residual urea clearance ≤ 2 mL/min, non-missing HD section	Prebiotics: high-amylose corn starch 15 g/day (*n* = 20)	Placebo (*n* = 20)	p-CS, IS hs-CRP	6 weeks
Viramontes-Hörner et al., 2015 [28]	Mexico	Randomized, double-blind, placebo-controlled clinical trial	42 (32/10)	40	23	HD thrice-weekly, at least 3 months	Synbiotics: B. lactis Bi-07 and L. acidophilus NCFM; prebiotic fiber (inulin) (*n* = 22)	Placebo (*n* = 20)	hs-CRP, IL-6, TNF-α	2 months
Xie et al., 2015 [29]	China	Randomized, placebo-controlled clinical trial	124 (68/56)	52	22	HD thrice-weekly, 4 h/session, Kt/V > 1.2 (1.46 ± 0.13)	Prebiotics: 10 g/d fiber (Group A, *n* = 41), 20 g/d (Group B, *n* =39)	Placebo (*n* = 44)	hs-CRP, IL-6, TNF-α, IL-8, TAC, GSH, MDA, SOD	6 weeks
Shariaty et al., 2017 [30]	Iran	Randomized, double-blind, placebo-controlled clinical trial	36 (20/16)	58	NA	HD thrice-weekly, 4 h/session	Probiotics: L. acidophilus, L. casei, L. rhamnosus, L. bulgaricus, B. breve, B. longum, S. thermophiles (500 mg/d) (*n* = 18)	Placebo (*n* = 18)	hs-CRP	3 months
Soleimani et al., 2017 [31]	Iran	Randomized, double-blind, placebo-controlled clinical trial	60 (40/20)	56	26	HD for ≥1 year, Kt/V probiotics group 1.38± 0.24, placebo group 1.35 ± 0.20	Probiotics: L. casei, L. acidophilus, and B. bifidum (*n* = 30)	Placebo (*n* = 30)	hs-CRP TAC, GSH, MDA	12 weeks
Borges et al., 2018 [32]	Brazil	Randomized, double-blind, placebo-controlled clinical trial	33 (21/12)	52	25	HD thrice-weekly, at least 6 months, 3−4.5 h/session	Probiotics: S. thermophilus, L. acidophilus, and B. longum (*n* = 16)	Placebo (*n* = 17)	p-CS, IS, IAA hs-CRP, IL-6	3 months
Eidi et al., 2018 [20]	Iran	Randomized, Triple-blind, placebo-controlled clinical trial	42 (32/10)	58	24	HD at least 3 months, hemodialysis 8.41 ± 4.14 h per week, Kt/V 1.43 ± 0.14	Probiotics: L. Rhamnosus (*n* = 21)	Placebo (*n* = 21)	p-CS, phenol	4 weeks
Esgalhado et al., 2018 [21]	Brazil	Randomized, double-blind, placebo-controlled clinical trial	31 (18/13)	55	26	HD for at least 6 months, Kt/V 1.4 ± 0.3	Prebiotics: resistant starch (16 g/d) (*n* = 15)	Placebo (*n* = 16)	p-CS, IS hs-CRP, IL-6	4 weeks
Khosroshahi et al., 2018 [33]	Iran	Randomized, double-blind, placebo-controlled clinical trial	44 (28/16)	56	23	Chronic hemodialysis	Prebiotics: high amylose resistant starch (HAM-RS2), 20 g/d (the first 4-weeks) and 25 g/d (the second 4-weeks) (*n* = 22)	Placebo (*n* = 22)	hs-CRP, IL-6, TNF-α, IL-1β TAC, MDA	8 weeks
Lopes et al., 2018 [34]	Brazil	Randomized, single-blind, placebo-controlled clinical trial	58 (38/20)	63	24	HD thrice weekly, 4 h/session	Synbiotics: 40 g of extruded sorghum + 100 mL of unfermented probiotic milk (*n* = 29)	40 g of extruded corn +100 mL of pasteurized milk (*n* = 29)	hs-CRP, IL-6, TNF-α, IL-10 TAC, MDA, SOD	7 weeks
Haghighat et al., 2019 [35]	Iran	Randomized, double-blind, placebo-controlled clinical trial	65 (34/31)	46	23	HD thrice weekly, at least 3 months, 3−4.5 h/session, mL/min, Kt/V 1.46 ± 0.28	Probiotics: L. acidophilus T16,B. lactis BIA-6, B. bifidum BIA 6 and B. longum LAF-5 (*n* = 23) Synbiotics: probiotics + prebiotics (*n* = 23)	Placebo (*n* = 19)	VCAM-1, CK18, ICAM-1	12 weeks
Khosroshahi et al., 2019 [36]	Iran	Randomized, double-blind, placebo-controlled clinical trial	50 (29/21)	55	24	HD thrice-weekly for at least 6 months	Prebiotics: high amylose resistant starch (HAM-RS2) 20 g/d (the first 4 weeks) and 25 g/d (the second 4 weeks) (*n* = 23)	Placebo (*n* = 21)	p-CS, IS hs-CRP TAC	8 weeks
Kooshki et al., 2019 [37]	Iran	Randomized, double-blind, placebo-controlled clinical trial	46 (21/25)	63	24	HD thrice-weekly, 4 h/session	Synbiotics: L. coagulans and fructo-oligosaccharides 100 mg, 2 tablets/d (*n* = 23)	Placebo (*n* = 23)	hs-CRP MDA	8 weeks
Laffin et al., 2019 [38]	Iran	Randomized, double-blind, placebo-controlled clinical trial	20 (13/7)	55	NA	NA	Prebiotics: high amylose resistant starch (HAM-RS2) 20 g/d (the first 4 weeks) and 25 g/d (the second 4 weeks) (*n* = 9)	Placebo (*n* = 11)	IL-6, TNFα	8 weeks
Lopes et al., 2019 [39]	Brazil	Randomized, single-blind, placebo-controlled clinical trial	58 (38/20)	63	24	HD thrice weekly, 4 h/session	Synbiotics: 40 g of extruded sorghum + 100 mL of unfermented probiotic milk (*n* = 29)	40 g of extruded corn +100 mL of pasteurized milk (*n* = 29)	p-CS, IS, IAA	7 weeks
Soleimani et al., 2019 [40]	Iran	Randomized, double-blind, placebo-controlled clinical trial	60 (42/18)	63	26	NA	Synbiotics: L. acidophilus, L. casei, and B. bifidum; prebiotic fiber(inulin) (*n* = 30)	Placebo (*n* = 30)	hs-CRP TAC, GSH, MDA	12 weeks
Azevedo et al., 2020 [22]	Brazil	Randomized, double-blind, placebo-controlled clinical trial	31 (18/13)	55	NA	NA	Prebiotics: resistant starch (16 g/d) (*n* = 15)	Placebo (*n* = 16)	IAA	4 weeks
Haghighat et al., 2020 [41]	Iran	Randomized, double-blind, placebo-controlled clinical trial	65 (34/31)	46	23	HD thrice weekly, at least 3 months, 3−4.5 h/session, Kt/V Synbiotics group 1.37 ± 0.29, Probiotics group 1.46 ± 0.25, Placebo 1.57 ± 0.28	Probiotics: L. acidophilus T16, B. bifidum BIA 6, B. longum LAF-5, and B. lactis BIA-6 (*n* = 23) Synbiotics: probiotics + prebiotics (*n* = 23)	Placebo (*n* = 19)	Endotoxin hs-CRP, IL-6	12 weeks
Liu et al., 2020 [24]	China	Randomized, double-blind, placebo-controlled clinical trial	50 (28/22)	48	20	HD for at least 3 months	Probiotics: B. longum, L. acidophilus, E. faecalis 210 mg, twice daily (*n* = 22)	Placebo (*n* = 23)	Endotoxin hs-CRP, IL-6, TNF-α, E-selectin, ICAM-1	6 months
Mirzaeian et al., 2020 [42]	Iran	Randomized, double-blind, placebo-controlled clinical trial	42 (30/12)	60	24.72	HD thrice weekly, at least 6 months, for no less then 4 h each time	Synbiotics: L. casei, L. acidophilus, L. rhamnosus, L. bulgaricus, B. breve, B. longum and S. thermophiles; fructo-oligosaccharide. Two synbiotic capsules daily (*n* = 21)	Placebo (*n* = 21)	IS, phenol hs-CRP	8 weeks
Paiva et al., 2020 [23]	Brazil	Randomized, double-blind, placebo-controlled clinical trial	16 (9/7)	55	26	HD for more than 6 months, Kt/V 1.4 ± 0.2	Prebiotics: resistant starch (16 g/d) (*n* = 8)	Placebo (*n* = 8)	IP-10, PDGF, RANTES	4 weeks
Lim et al., 2021 [25]	Taiwan	Randomized, double-blind, placebo-controlled clinical trial	50 (20/30)	59	24.5	HD thrice weekly, for at least 6 months, 4 h/session, Kt/V 1.79 ± 0.21 1.84 ± 0.20	Probiotics: Lactococcus lactis subsp. Lactis LL358, L. salivarius LS159, and L. pentosus LPE588 (*n* = 25)	Placebo (*n* = 25)	p-CS, IS, hs-CRP, IL-6, TNF-α	6 months

NA, not available; L., Lactobacillus; S., Streptococcus; B., Bifidobacterium; hs-CRP, high-sensitivity C-reactive protein; TIG, total indoxyl glucuronide; HD, hemodialysis; p-CS, p-cresyl sulfate; IS, indoxyl sulfate; IL-6, interleukin 6; TNF-α, tumor necrosis factor alpha; IL-8, interleukin 8; TAC, total antioxidant capacity; GSH, glutathione; MDA, malondialdehyde; SOD, superoxide dismutase; IAA, indole-3-acetic acid; IL-1β, interleukin 1 beta; IL-10, interleukin 10; VCAM-1, vascular cell adhesion molecule-1; CK18, cytokeratin18; ICAM-1, intercellular adhesion molecule-1; IP-10, interferon gamma-induced protein 10; PDGF, platelet-derived growth factor; RANTES, regulated on activation, normal T-Cell expressed and secreted.

## Data Availability

All data are reported in this manuscript.

## Appendix A

**Table A1:** Detailed search strategies for each database. MeSH terms, search terms, and combinations of the two were used for each database search.

Database	Detailed Search Strategies	Records Founded
MEDLINE/PUBMED	(“probiotics”(MeSH Terms) OR probiotics(Text Word) OR “prebiotics”(MeSH Terms) OR prebiotics(Text Word) OR “dietary fiber”(MeSH Terms) OR Dietary Fiber(Text Word) OR “resistant starch”(MeSH Terms) OR Resistant Starch(Text Word) OR “synbiotics”(MeSH Terms) OR synbiotics(Text Word)) AND (“renal dialysis”(MeSH Terms) OR hemodialysis(Text Word))	100
EMBASE	(‘probiotic agent’ OR ‘prebiotic agent’ OR ‘dietary fiber’ OR ‘resistant starch’ OR ‘synbiotic agent’) AND hemodialysis	231
Cochrane Library	(probiotics OR prebiotics OR “dietary fiber” OR “resistant starch” OR synbiotics) AND hemodialysis	97
Web of Science	(probiotics OR prebiotics OR “dietary fiber” OR “resistant starch” OR synbiotics) AND hemodialysis	112

Ultimately, 540 records were found, 100 from MEDLINE/PubMed, 231 from EMBASE, 97 from Cochrane Library, and 112 from the Web of Science. Studies were further selected according to the inclusion criteria listed in the Material and Methods (Figure 1).
